# The impact of frailty on survival in elderly intensive care patients with COVID-19: the COVIP study

**DOI:** 10.1186/s13054-021-03551-3

**Published:** 2021-04-19

**Authors:** Christian Jung, Hans Flaatten, Jesper Fjølner, Raphael Romano Bruno, Bernhard Wernly, Antonio Artigas, Bernardo Bollen Pinto, Joerg C. Schefold, Georg Wolff, Malte Kelm, Michael Beil, Sigal Sviri, Peter Vernon van Heerden, Wojciech Szczeklik, Miroslaw Czuczwar, Muhammed Elhadi, Michael Joannidis, Sandra Oeyen, Tilemachos Zafeiridis, Brian Marsh, Finn H. Andersen, Rui Moreno, Maurizio Cecconi, Susannah Leaver, Ariane Boumendil, Dylan W. De Lange, Bertrand Guidet, COVIP study group, Hans Flaatten, Bernhard Wernly, Antonio Artigas, Michael Beil, Sigal Sviri, Peter Vernon van Heerden, Wojciech Szczeklik, Muhammed Elhadi, Tilemachos Zafeiridis, Rui Moreno, Maurizio Cecconi, Ariane Boumendil, Hazem Maarouf Abosheaishaa, Enas M. Y. Abualqumboz, Abdullah Khudhur Ahmed, Hazem Ahmed, Zoi Aidoni, Cesar Aldecoa, Nica Alexandru, Yasmin Khairy NasrEldin Mohamed Ali, Mohammed Al-Sadawi, Kasper Andersen, Finn H. Andersen, Rui Assis, Mohammed A. Azab, Ahmed Y. Azzam, Mohamed Raafat Badawy, Ida Riise Balleby, Eberhard Barth, Eberhard Barth, Nawfel Ben-HAmouda, Guillaume Besch, Sebastien Besset, Anders Thais Bjerregaard, Helene Brix, Raphael Romano Bruno, Jens Brushoej, Helle Bundgaard, Philippe Burtin, Anais Caillard, Isabel Canas-Perez, Cyril Charron, Evangelia Chrisanthopoulou, Vittoria Comellini, Alex Cornet, Patricia Jimeno Cubero, Miroslaw Czuczwar, Stéphane Dauger, Cristina Diaz-Rodriguez, Willem Dieperink, Zouhir Dindane, Michel Djibré, Tom Dormans, Alexander Dullenkopf, Guillaume Dumas, Yumna A. Elgazzar, Philipp Eller, Ahmed Elsaka, Mirjam Evers, Andreas Faltlhauser, Aida Fernández Ferreira, Jesper Fjølner, Yvan Fleury, Arnaud Galbois, Pierre Garcon, Marc Garnier, Ryszard Gawda, Abdelilah Ghannam, Ulrich Goebel, Gemma Gomà, Bruno Goncalves, André Gordinho, Martijn Groenendijk, Emmanuel Guerot, Bertrand Guidet, Mohan Gurjar, Hendrik Haake, Lenneke Haas, Ayman Abdelmawgoad Habib, Michael Hahn, Maria Aagaard Hansen, Momin Majed Yousuf Hilles, Aliae A. R. Mohamed Hussein, David Iglesias, Michael Joannidis, Christian Jung, Igor Jurcisin, Hans-Joachim Kabitz, Malte Kelm, Detlef Kindgen-Milles, Jakub Klimkiewicz, Karl Friedrich Kuhn, Anselm Kunstein, Muhammed Kurt, Dylan W. De Lange, Susannah Leaver, Matthias Lutz, Ata Mahmoodpoor, Julien Maizel, Nathalie Marin, Brian Marsh, Buno Megarbane, Dieter Mesotten, Patrick Meybohm, Christian Meyer, Angela Prado Mira, Silvio a Namendys-Silva, Helene Korvenius Nedergaard, Saad Nseir, Sandra Oeyen, Theresa Olasveengen, Ana Isabel Pinho Oliveira, Johanna Oziel, Antonios Papadogoulas, David Perez-Torres, Bernardo Bollen Pinto, Gaël Piton, Gaëtan Plantefeve, Tudor Poerner, Jesús Priego, Ahmed Rabha, Winfried Randerath, Jean-herlé Raphaelen, Pascal Reper, Jean-Philippe Rigaud, Susana Arias Rivera, Andrea Roberti, Luis Romundstad, Nikoletta Rovina, Rehab Salah, Mahmoud Saleh, Susana Sancho, Maria de Lurdes Campos Santos, Henrique Santos, Stefan Schaller, Joerg C. Schefold, Michael Schuster, Gonxhe Shala, Britt Sjøbø, Stephan Steiner, Hans Frank Strietzel, Sigal Sviri, Walter Swinnen, Luis Tamayo-Lomas, Samar Tharwat, Teresa Tomasa, Stine Uhrenholt, Marie Vaissiere, Arnaud Valent, Xavier Valette, Thierry Vanderlinden, Eric Mayor Vázquez, Mercedes Ibarz Villamayor, Maja Villefrance, Ingo Voigt, Kyrillos Wassim, Martin Welte, Georg Wolff, Jakob Wollborn, Begoña Zalba-Etayo, Marieke Zegers

**Affiliations:** 1 Department of Cardiology, Pulmonology and Vascular Medicine, Medical Faculty, Heinrich-Heine-University Duesseldorf, Moorenstraße 5, 40225 Duesseldorf, Germany; 2 Department of Clinical Medicine, University of Bergen, Bergen, Norway; 3 Department of Anaesthesia and Intensive Care, Haukeland University Hospital, Bergen, Norway; 4 Department of Intensive Care, Aarhus University Hospital, Aarhus, Denmark; 5 Department of Cardiology, Paracelsus Medical University, Salzburg, Austria; 6 Department of Intensive Care Medicine, CIBER Enfermedades Respiratorias, Corporacion Sanitaria Universitaria Parc Tauli, Autonomous University of Barcelona, Sabadell, Spain; 7 Department of Acute Medicine, Geneva University Hospitals, Geneva, Switzerland; 8 Department of Intensive Care Medicine, Inselspital, Universitätsspital, University of Bern, Bern, Switzerland; 9 Department of Medical Intensive Care, Hadassah University Medical Center, Jerusalem, Israel; 10 General Intensive Care Unit, Hadassah University Medical Center, Jerusalem, Israel; 11 Center for Intensive Care and Perioperative Medicine, Jagiellonian University Medical College, Krakow, Poland; 12 2nd Department of Anesthesiology and Intensive Care, Medical University of Lublin, Staszica 16, 20-081 Lublin, Poland; 13 Faculty of Medicine, University of Tripoli, Tripoli, Libya; 14 Division of Intensive Care and Emergency Medicine, Department of Internal Medicine, Medical University Innsbruck, Innsbruck, Austria; 15 Department of Intensive Care 1K12IC, Ghent University Hospital, Ghent, Belgium; 16 Intensive Care Unit General Hospital of Larissa, Larissa, Greece; 17 Mater Misericordiae University Hospital, Dublin, Ireland; 18 Department of Anaesthesia and Intensive Care, Ålesund Hospital, Ålesund, Norway; 19 Department of Circulation and Medical Imaging, Norwegian University of Science and Technology, Trondheim, Norway; 20 Unidade de Cuidados Intensivos Neurocríticos e Trauma, Hospital de São José, Centro Hospitalar Universitário de Lisboa Central, Faculdade de Ciências Médicas de Lisboa, Nova Médical School, Lisbon, Portugal; 21 Department of Anaesthesia, IRCCS Instituto Clínico Humanitas, Humanitas University, Milan, Italy; 22 General Intensive Care, St George’s University Hospitals NHS Foundation Trust, London, UK; 23 Sorbonne Universités, UPMC Univ Paris 06, INSERM, UMR_S 1136, Institut Pierre Louis d’Epidémiologie et de Santé Publique, Equipe: épidémiologie hospitalière qualité et organisation des soins, 75012 Paris, France; 24 Assistance Publique - Hôpitaux de Paris, Hôpital Saint-Antoine, service de réanimation médicale, 75012 Paris, France; 25 Department of Intensive Care Medicine, University Medical Center, University Utrecht, Utrecht, The Netherlands

**Keywords:** COVID-19, Frailty, Outcome, Elderly, Pandemia

## Abstract

**Background:**

The COVID-19 pandemic has led highly developed healthcare systems to the brink of collapse due to the large numbers of patients being admitted into hospitals. One of the potential prognostic indicators in patients with COVID-19 is frailty. The degree of frailty could be used to assist both the triage into intensive care, and decisions regarding treatment limitations. Our study sought to determine the interaction of frailty and age in elderly COVID-19 ICU patients.

**Methods:**

A prospective multicentre study of COVID-19 patients ≥ 70 years admitted to intensive care in 138 ICUs from 28 countries was conducted. The primary endpoint was 30-day mortality. Frailty was assessed using the clinical frailty scale. Additionally, comorbidities, management strategies and treatment limitations were recorded.

**Results:**

The study included 1346 patients (28% female) with a median age of 75 years (IQR 72–78, range 70–96), 16.3% were older than 80 years, and 21% of the patients were frail. The overall survival at 30 days was 59% (95% CI 56–62), with 66% (63–69) in fit, 53% (47–61) in vulnerable and 41% (35–47) in frail patients (*p* < 0.001). In frail patients, there was no difference in 30-day survival between different age categories. Frailty was linked to an increased use of treatment limitations and less use of mechanical ventilation. In a model controlling for age, disease severity, sex, treatment limitations and comorbidities, frailty was independently associated with lower survival.

**Conclusion:**

Frailty provides relevant prognostic information in elderly COVID-19 patients in addition to age and comorbidities.

*Trial registration* Clinicaltrials.gov: NCT04321265, registered 19 March 2020.

**Supplementary Information:**

The online version contains supplementary material available at 10.1186/s13054-021-03551-3.

## Introduction

The first wave of the SARS-CoV-2 coronavirus disease (COVID-19) pandemic disproportionally affected the elderly population, creating an unprecedented influx of patients into hospital and intensive care [1]. Consequently, ICU capacity had to be increased several fold [2]. Despite this, many countries struggled with limited resources and were forced into a stricter admission policy to ICUs. For various reasons, this disproportionately affected the very old subgroup of patients, with reports of ad hoc criteria used for ICU admission, some even based on age alone.

Over the last few years, the assessment of frailty at admission to the ICU has become increasingly popular. The clinical frailty scale (CFS) proved to be a useful tool in predicting the chances of ICU survival in very old intensive care patients [3–5] and is one of a number of tools commonly used to assess frailty. In previous studies, the CFS demonstrated a high inter-rater reliability [6].

Indeed, the UK National Institute for Health and Care Excellence (NICE) advocates the use of the CFS in clinical decision making for patients with COVID-19 ≥ 65 years [7]. Additionally, during the present COVID-19 pandemic frailty has been found to be strongly associated with all-cause mortality risk in hospitalised older patients [8, 9]. Hewitt et al. showed in a study of 1564 adult (> 18 years) COVID-19 patients that frailty was a better tool for risk stratification than age or concomitant diseases [10]. In another, smaller study of 677 older (> 65 years) patients with COVID-19, frailty was associated with mortality after a mean follow-up of 34 days [8]. A similar association between frailty and hospital mortality was shown in a smaller cohort of 42 COVID-19 patients [9]. Bellelli et al. also demonstrated in a cohort of 105 patients that frailty could be used for risk evaluation of COVID-19 and proposed the systematic collection of frailty in all patients at hospital admission [11]. By contrast, recently published retrospective studies focusing on non-ICU COVID-19 patients found that frailty had no or low diagnostic or prognostic value [12, 13]. There is therefore need for more high-quality data on patient outcomes to determine whether frailty, as assessed by the CFS, and in particular its interaction with age can be used for prognostication in critically ill elderly patients with COVID-19 [14]. This is of paramount importance in order to establish an ethical and medically appropriate rationing of ICU care.

The main aim of the present study was to study the outcome of elderly patients with COVID-19 admitted to an intensive care unit and to study the influence of frailty on outcome.

## Methods

### Design and setting

This is a prospective multicentre study of COVID-19 patients ≥ 70 years old admitted to the ICU. Recruitment took place from 19 March to 26 May 2020, in 138 intensive care units in 28 countries (for a list of collaborators, see Additional file 1, for a map of participating ICUs and patients included see Additional file 2). The study was planned and conducted by the very old intensive care patients (VIP) project within the European Society of Intensive Care Medicine (ESICM) (www.vipstudy.org) who also endorsed the study. National coordinators were responsible for the recruitment of ICUs, coordinated national and local ethical permission and supervised patient recruitment at the national level. Ethical approval was mandatory for study participation in each country. Due to the diversity of ethical consent procedures, some countries could recruit patients without informed consent while the rest had to obtain it. The study deliberately allowed for co-enrolment of study patients to additional observational COVID-19 studies. To limit workload, screening failures for the study were not recorded. The study was registered on ClinicalTrials.gov (ID: NCT04321265) and adhered to the European Union General Data Privacy Regulation (GDPR) directive, which is implemented in most participating countries.

It was agreed that the first period of the COVIP study would stop on 26th of May 2020 corresponding with the slowing down of the first wave of critically ill patients in most of the participating countries. However, the study continued to recruit in order to catch a possible second wave. The present study included patients from the first recruitment period. Each participating ICU included consecutive patients up to and including those admitted on 26th of May 2020. COVID-19 diagnosis was based on a positive polymerase chain reaction (PCR) test. Patients were followed up until death, 30 days, and three months after ICU admission. Similar to the previous VIP studies, a website was set up to facilitate dissemination of information about the study and to allow for data entry using an electronic case report form (CRF).

### Study population

Patients who were 70 years or older with proven COVID-19 and admitted to an ICU were eligible. Pre-ICU triage was not a part of this study. To avoid duplication caused by the transfer of a patient from one ICU to another, each patient could only be entered once into the database regardless of readmission, transfer or other reason. This resulted in a single electronic CRF per patient. The reference date was day 1 of the first admission to an ICU. All consecutive dates were numbered sequentially from the admission date.

### Data collection

Centres collected the data using a uniform online CRF. The sequential organ-failure assessment (SOFA) score on admission was calculated either manually or using an online calculator in the electronic CRF as described previously [3, 4]. Additionally, the first arterial blood gas analysis with pO_2_ [mmHg] and the FiO_2_ [%] to calculate the PaO_2_/FiO_2_ index (pO_2_/FiO_2_ ratio) on admission was recorded. Length of stay (LOS) was recorded in hours. As described previously, [3] the electronic CRF and database ran on a secure server set up by and stored at Aarhus University, Aarhus, Denmark.

### Frailty and comorbidities

The frailty level prior to the acute illness and hospital admission was assessed using the clinical frailty scale (CFS). This is an intuitive pictographic description along with information required to perform the assessment [15]. The CFS defines nine classes from very fit to terminally ill (Additional file 3). The required information could be obtained either from the patient, the caregiver/family or hospital records [4, 16]. We used the English version of the CFS. Patients with a CFS of 1–3 were classified as fit, those with a CFS of 4 as vulnerable and a CFS of 5 or higher as frail. CFS assessment was performed as described previously with excellent inter-rater variation [4]. The definitions of pre-existing comorbidities are provided in Additional file 4.

### Outcome measurement

The primary endpoint was the survival status assessed at 30 days after ICU admission. The outcome at 90 days was also assessed. Data could be retrieved either directly, from the hospital administration system or following telephone follow-up. Limitation of life-sustaining therapies such as withholding or withdrawing organ support was documented based on international recommendations [17] although there is a large variation in Europe in the use of end-of-life care [16]. The definitions of organ support are detailed in Additional file 5.

### Statistical analysis

No formal sample size calculation prior to this purely observational study was performed. The analysis plan was finalised prior to any analysis. The primary exposure was frailty (fit, vulnerable or frail at ICU admission), the primary outcome was 30-day survival, and the secondary outcomes were overall survival up to 90 days after ICU admission, organ support (vasoactive drugs, mechanical ventilation, non-invasive ventilation and renal replacement therapy) and treatment limitation. Group comparisons for continuous variables were performed using the Kruskal–Wallis test if no-normally distributed, and ANOVA if normally distributed; for categorical variables the Chi square test was used. Overall survival from ICU admission was estimated using the Kaplan–Meier method. If lost to follow-up at 90 days, patients were censored at 30 days or ICU discharge if status at 30 days was unknown. Survival between groups was compared using the log-rank test. Incidence of organ support and treatment limitation were estimated using cumulative incidence analysis considering ICU death and ICU discharge as competing risks. Comparisons between groups were performed using Gray’s test.

Multivariate analysis of primary and secondary outcomes: to account for the multilevel structure of the data with individuals nested into the ICU, all multivariate models were built including a random intercept by ICU, assuming a Gaussian distribution for the random effect. The random effect was tested by comparing log-likelihood of two models including frailty with and without random effect.

Three sequential random effects, multilevel Cox regression models, were used to evaluate the impact of frailty on both 30-day and 90-day survival. First, we estimated the impact of frailty on outcome without adjustment for confounding using a baseline model including only frailty (model 1). Second, to estimate the impact of frailty when adjusting for patients’ baseline characteristics, the following covariates were added to model 1: age (as a continuous variable), sex, comorbidities, SOFA score, BMI, PaO_2_/FiO_2_ (as continuous variables). Third, to evaluate whether the effect of frailty was independent of ICU management strategies, both organ support and treatment limitation (model 3) were added to model 2 as time-dependent covariates (variables start at 0 for all subjects and are recoded to 1 only when organ support is received or when limitation occurs). Two sequential random effects, multilevel cause-specific Cox regression models, were used to evaluate the impact of frailty on organ support and treatment limitation. First, we estimated the impact of frailty on variable of interest without adjustment for confounding using a baseline model including only frailty (model 1). Second, to estimate the impact of frailty when adjusting for patients’ baseline characteristics the following covariates were added to model 1: age (as a continuous variable), sex, comorbidities, SOFA score, BMI, PaO_2_/FiO_2_ (as continuous variables).

A sensitivity analysis was conducted to investigate whether results differ including only European patients.

All p values were two-sided, and *p* < 0.05 was considered statistically significant. Statistical analyses were performed with R 3.2.3 software packages (R Development Core Team, Vienna, Austria).

## Results

This study included 1346 patients from 138 ICUs across 28 countries. The median number of recruited patients per ICU was 7 (IQR 3–12). The study flow chart is illustrated in Additional file 6. Survival at 30 and 90, respectively, was available in 97% and 90% of the cohort.

Frailty was assessed by an ICU physician in 55%, by dedicated research staff in 21%, by an ICU nurse in 13%, and by other personnel in 11% of the cases. Further information for CFS assessment was provided by hospital records (51%), the family or caregivers (25%), by the patient (22%) and by other information (2%). Patients characteristics and outcome were similar whether CFS was rated by an ICU physician or other personal and whether the CFS was based on hospital records or other sources.

The median age of patients was 75 years (IQR 72–78, range 70–96. Median CFS was 3 (IQR 2–4) and 20.7% of the patients were frail (CFS ≥ 5). Only one patient had CFS 9 so this group was not further split. Further baseline characteristics of the study population are given in Table 1.

**Table 1:** Patient characteristics of the study population

	All patients	Fit (CFS 1–3)	Vulnerable (CFS: 4)	Frail (CFS: 5–9)
	*n* = 1346	*n* = 874	*n* = 193	*n* = 279
Female sex				
* n* (%)	381 (28)	209 (24)	64 (34)	108 (39)
Age				
Median (IQR)	75 (72–78)	74 (72–77)	76 (73–79)	78 (74–82)
Frailty score—CFS				
Median (IQR)	3 (2–4)	2 (2–3)	4 (4–4)	6 (5–7)
SOFA score				
Median (IQR)	6 (3–8)	5 (3–8)	6 (3–8)	6 (4–9)
BMI				
Median (IQR)	28 (25–31)	27 (25–30)	28 (25–31)	28 (24–31)
PaO_2_/FiO_2_ (mmHg), n (%)				
≤ 100	485 (37)	319 (37)	65 (35)	101 (37)
> 100–200	546 (41)	376 (43)	76 (40)	94 (34)
> 200–300	173 (13)	111 (13)	25 (13)	37 (13)
> 300	125 (9)	59 (7)	11 (12)	44 (16)
Comorbidities, n (%)				
Diabetes mellitus	471 (35)	240 (27)	89 (47)	142 (51)
Ischemic heart disease	291 (22)	127 (14)	59 (31)	105 (39)
Chronic renal insufficiency	211 (16)	77 (9)	31 (17)	103 (38)
Arterial Hypertension	896 (67)	529 (61)	139 (73)	228 (82)
Pulmonary disease	314 (24)	169 (20)	60 (32)	85 (31)
Chronic heart failure	205 (15)	72 (6)	49 (26)	84 (32)

*CFS* clinical frailty scale, *SOFA* sequential organ failure assessment score, *IQR* inter-quartile range, *BMI* body mass index, *PaO*_*2*_ partial pressure of oxygen, *FiO*_*2*_ fraction of inspired oxygen

**Fig. 1:**
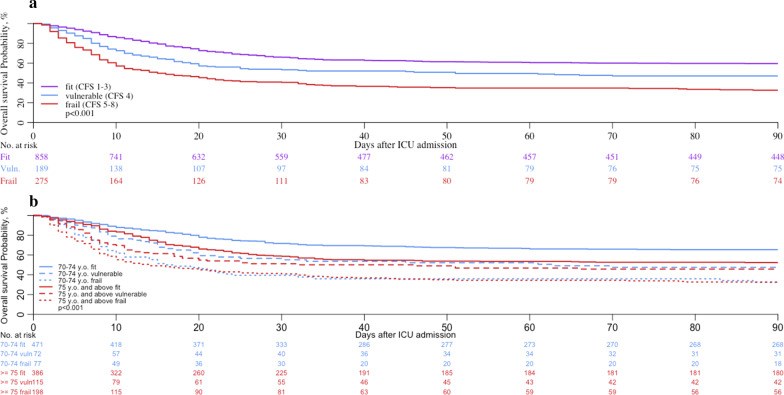
**a** Kaplan–Meier curve illustrating survival dependent on clinical frailty scale (CFS) category: fit, vulnerable and frail. **b** Patients were divided according to the age median (75 years) and survival was illustrated according to their frailty category

The overall survival at 30 days was 59% (95% CI 56–62). Ninety-day survival was 52% and decreased with increasing CFS as illustrated in Additional file 7. Numbers of deaths are reported in Additional file 8. Figure 1a shows the prognostic relevance of frailty in a survival analysis. Figure 1b illustrates the frailty category by age groups below and above 75 years. Of note, there is no difference between age groups in frail patients.

Survival at 30 days was 66% (63–69) in fit, 53% (47–61) in vulnerable and 41% (35–47) in frail patients. The difference persisted at 90 days with respective survival of 59% (56–63), 47% (40–55) and 33% (27–39) *p* < 0.001). Length of ICU stay for patients discharged alive was 15 days (IQR7.0–29.0) in fit, 10 days (IQR 5–21) in vulnerable and 6 days (IQR 3.0–13) in frail patients (*p* < 0.001).

**Fig. 2:**
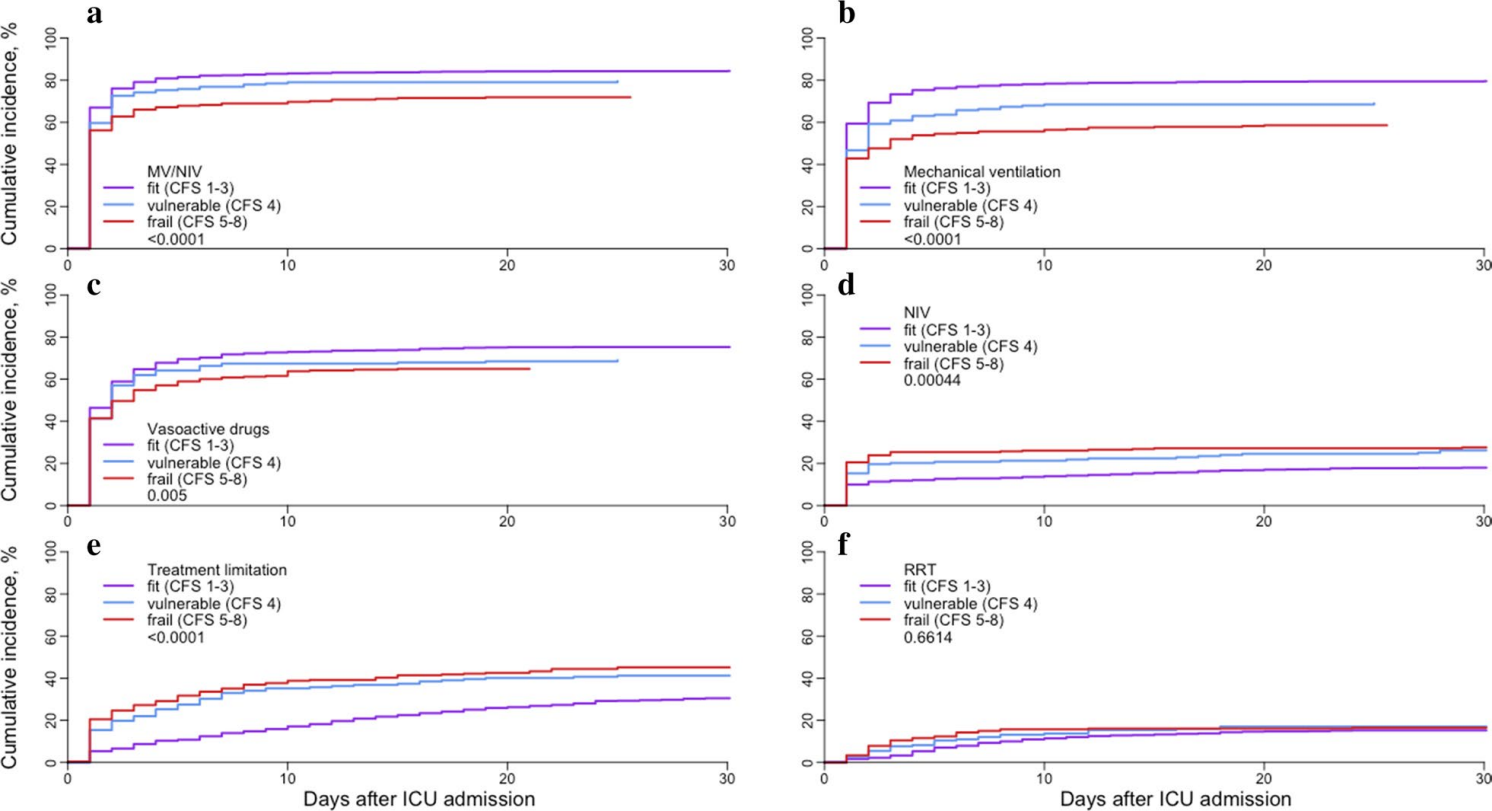
Cumulative incidence of organ support and treatment limitations. **a** Combined mechanical ventilation (MV) and non-invasive ventilation. **b** Mechanical ventilation (MV). **c** Vasoactive drugs. **d** Non-invasive ventilation (NIV). **e** Treatment limitations. **f** Renal replacement therapy (RRT)

Table 2 shows models revealing the association between frailty and outcome even after controlling for comorbidities and disease severity and treatment strategies. Frailty was associated with increased use of treatment limitations and reduction in respiratory support as shown in Fig. 2 (cumulative incidences in Additional file 9). The use of treatment limitations was significantly higher in frail patients compared to fit patients and vulnerable patients (20-day cumulative incidence was 26% (95% CI 23–29) for fit patients, 40% (33–47) for vulnerable patients and 43% (95% CI 37–48) for frail patients (*p* < 0.001). The association between frailty and treatment limitation remained statistically significant after adjustment for patient characteristics (aHR for frail vs fit patients 2.79 (95% CI 1.96–3.91, *p* < 0.001)).

**Table 2:** Three sequential random effects and multilevel Cox regression models were used to evaluate the impact of frailty on both 30-days survival

	Model 1 unadjusted HR (95% CI)	*p* value	Model 2 adjusted HR (95% CI)	*p* value	Model 3 adjusted HR (95% CI)	*p* value
Survival at 30 days						
Vulnerable versus fit	1.75 (1.35–2.25)	< 0.001	1.55 (1.14–2.10)	0.011	1.14 (0.79–1.65)	0.4811
Frail versus fit	3.20 (2.56–4.13)	< 0.001	2.41 (1.77–3.27)	< 0.001	1.86 (1.36–2.52)	< 0.001
Treatment limitation						
Vulnerable versus fit	2.26 (1.73–2.96)	< 0.001	1.7 (1.21–2.38)	0.0021		
Frail versus fit	3.98 (3.08–5.21)	< 0.001	2.79 (1.96–3.91)	< 0.001		
Mechanical ventilation						
Vulnerable versus fit	0.83 (0.67–1.01)	0.055	0.92 (0.73–1.16)	0.5		
Frail versus fit	0.75 (0.62–0.92)	0.005	0.69 (0.54–0.87)	0.0043		
Non-invasive ventilation						
Vulnerable versus fit	1.58 (1.11–2.25)	0.011	1.22 (0.79–1.88)	0.37		
Frail versus fit	1.58 (1.12–2.24)	0.009	1.26 (0.8–1.95)	0.32		
Non-invasive ventilation/mechanical ventilation						
Vulnerable versus fit	0.94 (0.78–1.13)	0.51	1 (0.81–1.24)	0.99		
Frail versus fit	0.84 (0.7–1.02)	0.069	0.74 (0.58–0.91)	0.0096		
Vasoactive drugs						
Vulnerable versus fit	0.99 (0.81–1.22)	0.93	1.09 (0.86–1.39)	0.47		
Frail versus fit	1.01 (0.84–1.25)	0.88	0.9 (0.7–1.15)	0.44		
Renal replacement therapy						
Vulnerable versus fit	1.23 (0.81–1.86)	0.33	1.14 (0.7–1.87)	0.61		
Frail versus fit	1.62 (1.1–2.45)	0.014	1.01 (0.59–1.65)	0.98		

First, we estimated the impact of frailty on outcome without adjustment on confounding using a baseline model including only frailty (model 1). Second, to estimate the impact of frailty when adjusting on patients’ baseline characteristics the following covariates were added to model 1: age, sex, comorbidities, SOFA score, BMI, PaO_2_/FiO_2_. Third, to evaluate whether the effect of frailty was independent of ICU management strategies, both organ support and treatment limitation (model 3) were added to model 2 as time-dependent covariates. For all outcomes, significance of the random centre effect was tested comparing the likelihood of two models including frailty with and without random effect. Random effect was significant for all outcomes. No violation of the proportional hazard assumption was detected in the models

A sensitivity analysis showed that the findings were similar when excluding patients from outside Europe (*N* = 158).

## Discussion

Our study reveals three important findings in COVID-19 patients. First, frailty is a useful tool to stratify the risk of death at one and three months after admission to the ICU, and frailty offers an important additional prognostic information to the age in patients aging 70 and older. Second, outcome in terms of mortality in patients with a frailty level ≥ 5 is similar in patients across all age groups ≥ 70 years. Third, frailty was also associated with less use of mechanical ventilation and a higher rate of treatment limitation.

Age is frequently associated with a higher rate of hospitalisation, ICU admission and mortality in COVID-19 patients [18–20]. The high risk of mortality in older patients together with constraints on ICU bed availability may raise the question of rationing ICU admissions. Age alone should not be used and may be considered ageist [16]. For this reason, other factors should be investigated. Frailty as assessed by the CFS is a good candidate as it has previously been found to be strongly associated with mortality and is easy to use in acutely ill patients.

Before the COVID-19 pandemic emerged, frailty was already established as an important factor for outcome, particularly in very old ICU patients. This was documented in large studies from Canada [21] and Europe, and also in a systematic review. As a result, frailty was suggested early on during the pandemic as a useful tool to assist guiding therapy. In the UK, NICE issued guidelines advocating the use of the CFS in patients above 65 years to assist with decision making regarding ICU admission. Here, scores of five and above were thought to represent a worse prognosis in critically ill patients [22]. However, the evidence for using frailty was extrapolated from pre-pandemic data [23], and as a result, this guideline was heavily criticised for being based on insufficient data.

During this pandemic, there have been many discussions about the care of the old and the very old critical ill patients. Frailty has been the focus in four studies. In a retrospective single-centre study from Italy with 105 patients, the frailty index was found to be an independent predictor not only of in-hospital mortality but also for ICU admission. In another single-centre study from the UK of 215 hospitalised, non-ICU, patients both CFS and age were associated with mortality [24]. By contrast, in a larger study of 1071 hospitalised patients with COVID-19, CFS was not associated with mortality. To date, the largest investigation of frailty in COVID-19 patients is a multicentre observational study from the UK and Italy involving 1564 patients from 11 hospitals. The study included all hospitalised patients ≥ 18 years admitted with COVID-19 during a defined period and therefore differs from the present study which included only patients ≥ 70 years. They found a large proportion of patients were frail (CSF ≥ 5 in 51.4%) and that disease outcome was better predicted by frailty, measured with the CFS, than either age or comorbidity alone.

The importance of chronological age in COVID-19 has been extensively documented. In a retrospective case series, 1591 consecutive patients with a median age of 63 years were admitted to 72 Italian hospitals between February and March 2020 [18]. They found that patients with a median age of 63 years or more had a higher mortality than younger patients; they also required mechanical ventilation more frequently. This result was supported by another retrospective study from Germany in 10,021 hospitalised adult patients from 920 different hospitals [25]. Overall, patients aged 80 years or older had the highest mortality of 72%. These two studies, however, focused on chronological age alone with no frailty assessment used for outcome prognostication.

The use of frailty in general, and CFS in particular, was the focus of a recent editorial in ICM published prior to the pandemic [26]. This suggested that risk stratification should not be based on age alone but should include a frailty assessment. They also stressed the role of putting in place a time limited trial of treatment on admission to ICU as mortality after ICU treatment in frail elderly patients remains high. Current and recent research has not proven the long-term benefit of frailty assessment in these patients. The role of frailty may therefore better inform best interest decisions—i.e. whether burdens of ICU are more likely to outweigh benefits (or vice versa) but caution should be applied in excluding patients for ICU based on age/frailty status alone as there may be some patients denied ICU that still have the potential to benefit despite being frail.

The present research has several strengths [27]. It is a multicentre trial that recruited patients from 28, mainly European, countries. It included different types of hospitals thus reflecting diverse health care systems underlining the validity of the results. High quality data were collected prospectively despite the strain on health care systems during the pandemic. In addition, this study focuses exclusively on elderly patients who were admitted to an intensive care unit.

Our study, however, has a number of limitations: (1) No data was collected about the pre-ICU triage process and as a result we do not know how many very old critically ill patients were denied ICU-admission. (2) it is an unblinded study as it is difficult to conduct a blinded study for frailty. (3) CFS is not a suitable tool to evaluate patients with either temporary disability (For example as a result of trauma or delirium) or stable long-term disabilities (for example, cerebral palsy), learning disability or autism; in the inclusion and exclusion criteria of our cohort these considerations were not explicitly acknowledged, which may have promoted selection bias. Furthermore, there are other health conditions, unrelated to frailty, that can limit activity that might lead to an artificially high CFS that does not reflect true frailty. (4) Another limitation of this study is the lack of functional outcome data. While we were able to investigate associations with mortality, the extent of morbidity in survivors remains unclear. (5) No younger patients were included in this study for comparison. (6) The list of co-morbidities recorded was incomplete as only the most prevalent were documented, other comorbidities such as haematologic disorders or those with immune deficiencies were not recorded. (7) It was not possible to assess frailty in 7% of patients due to insufficient information. (8) We did not record information about the ethnic background, although it might be a potential confounding factor [28].

Our study does throw up some ethical dilemmas. There is an inter-relationship between high frailty scores and the unconscious bias of health care providers. For example, a high CFS on admission could lead the ICU health care provider to treat a patient less aggressively and to set a limitation of therapy earlier in their illness. Thus, the knowledge of frailty implicitly influences the outcome of the patient. We raise this as a limitation of our study similar to all studies describing outcomes in very old patients [10, 11, 24, 29], but also as a "caveat" for future studies. On the other hand, we know that frailty is the common, multifactorial endpoint of life, and therefore the presence of frailty per se (independent of its measurement) influences patient outcome and is thus a self-fulling prophecy.

## Conclusion

Frailty provides relevant prognostic information in elderly COVID-19 patients in addition to age and comorbidities. Therefore, we recommend that a frailty assessment should be routinely performed in these patients. In times of limited resources on the ICU, a frailty assessment of elderly patients could be included in a holistic assessment of patients.

## Supplementary Information

**Additional file 1.**: List of collaborators: COVIP-study; Description: List of COVIP study collaborators with affiliations

**Additional file 2.**: COVIP Country map; Distribution of study sites and included patients per country. The first number is the number of ICUs per country, the second the total number of included patients per country

**Additional file 3.**: Clinical Frailty Scale; Description: Pictograms and description of the Clinical Frailty Scale

**Additional file 4.**: Definition of the comorbidities; Description: Detailed definition of the comorbidities of patients included in the COVIP study

**Additional file 5.**: Definition of organ support; Description: Definition of organ support in recruited patients to be documented in the case report form

**Additional file 6.**: Consort flow chart; Description: Consort flow chart illustrating screening and inclusion into the COVIP study

**Additional file 7.**: Kaplan Meier curve illustrating survival dependent on clinical frailty scale (CFS); Description: Kaplan Meier curve illustrating survival dependent on clinical frailty scale (CFS) for each category

**Additional file 8.**: Numbers of deaths; Description: Numbers of deaths reported during the study/follow-up

**Additional file 9.**: Survival estimates for the primary endpoint (30-day mortality) and additional time points for fit, vulnerable and frail patients as well as cumulative incidence of treatment limitations and treatment modalities Description: Table of survival estimates for the primary endpoint (30-day mortality) and additional time points

## Data Availability

Individual participant data that underlie the results reported in this article are available to investigators whose proposed use of the data has been approved by the COVIP steering committee.

## Take-home-message

This prospective, large, international multicentre COVIP-study delineates the value of using frailty in the prognostication of elderly patient with COVID-19 up to three months after ICU admission. CFS is independently associated with outcome.

## Tweet

CFS is a valuable tool to assist prognostication in elderly ICU patients during the COVID-19 pandemic.
