# Supplementary material for: The impact of frailty on survival in elderly intensive care patients with COVID-19: the COVIP study
Source: Crit Care. 2021 Apr 19;25:149. doi: 10.1186/s13054-021-03551-3 (PMC8054503; doi:10.1186/s13054-021-03551-3)
Supplement: Supplementary file 1 — Additional file 1.: List of collaborators: COVIP-study; Description: List of COVIP study collaborators with affiliations [file 13054_2021_3551_MOESM1_ESM.docx]

**List of collaborators: COVIP-study**

| Hospital | City | ICU | Name |
| --- | --- | --- | --- |

**Austria**

| Medical University Graz | Graz | Allgemeine Medizin Intensivstation | Philipp Eller |
| --- | --- | --- | --- |
| Medical University Innsbruck | Innsbruck | Division of Intensive Care and Emergency Medicine, Department of Internal Medicine | Michael Joannidis |

**Belgium**

| Ziekenhuis Oost-Limburg | Genk | Department of Intensive Care | Dieter Mesotten |
| --- | --- | --- | --- |
| CHR Haute Senne | Soignies | Department of Intensive Care | Pascal Reper |
| Ghent University Hospital | Ghent | Department of Intensive Care | Sandra Oeyen |
| AZ Sint-Blasius | Dendermonde | Department of Intensive Care | Walter Swinnen |

**Denmark**

| **Hospital** | **City** | **ICU name** | **Contact name** |
| --- | --- | --- | --- |
| Herlev og Gentofte Hospital | Herlev | Intensiv Behandling | Helene Brix |
| Slagelse | Slagelse | Intensiv | Jens Brushoej |
| Regionshospitalet Horsens | Horsens | Intensiv | Maja Villefrance |
| Odense University Hospital | Odense | Intensive Care Unit | Helene Korvenius Nedergaard |
| Sygehus Lillebælt | Kolding | Intensiv | Anders Thais Bjerregaard |
| Regionshospitalet Viborg | Viborg | Intensiv | Ida Riise Balleby |
| Sygehus Sønderjylland | Aabenraa | Department of Anaesthesia and Intensive Care | Kasper Andersen |
| Regionshospitalet Herning | Herning | Intensiv Afdeling | Maria Aagaard Hansen |
| Nordsjællands Hospital | Hillerød | Department of Anaesthesia and Intensive Care | Stine Uhrenholt |
| Regionshospitalet Randers | Randers | Intensiv | Helle Bundgaard |
| Aarhus University Hospital | Aarhus | Department of Intensive Care | Jesper Fjølner |

**Egypt**

| Assiut University Hospital | Assiut | Medical ICU and Isolation Centers | Aliae AR Mohamed Hussein |
| --- | --- | --- | --- |
| One day surgery hospital | Nasr city | Cardiology ICU | Rehab Salah |
| Minia University Hospitals | Minia | MICU | Yasmin Khairy NasrEldin Mohamed Ali |
| Quweisna central hospital | Quweisna | MICU | Kyrillos Wassim |
| Mayo Isolation Hospital | Cairo Governorate | MICU | Yumna A. Elgazzar |
| Temi El amdid | Mansoura | Mansoura university Hospital | Samar Tharwat |
| Alazhar University Hospitals | Cairo |  | Ahmed Y. Azzam |
| one day surgery | nasr city | intermediate ccu | Ayman abdelmawgoad habib |
| Mostafa Mahmoud Specialized Hospital | Giza | MICU | Hazem Maarouf Abosheaishaa |
| Kar Al-Ainy Cairo University Hospital | Cairo | Sherif Mokhtar Cairo University ICU | Mohammed A Azab |

**England**

| St George´s University Hospitals NHS Foundation trust | London | General Intensive care | Susannah Leaver |
| --- | --- | --- | --- |

**France**

| Hôpital Privé Claude Galien | Quincy sous Sénart | Medico-surgical ICU | Arnaud Galbois |
| --- | --- | --- | --- |
| Saint Antoine | Paris | Medical intensive care unit | Bertrand Guidet |
| Hôpital Ambroise Paré | Boulogne Billancourt | Medical intensive care unit | Cyril Charron |
| Hopital Européen Georges Pompidou | Paris | Medical intensive care unit | Emmanuel Guerot |
| CHU de Besançon | Besançon | Medico-surgical ICU | Guillaume Besch |
| Dieppe General Hospital | Dieppe | Medical intensive care unit | Jean-Philippe Rigaud |
| CHU Amiens | Amiens | Medical intensive care unit | Julien Maizel |
| Tenon | Paris | Medico-surgical ICU | Michel Djibré |
| Clinique Du Millenaire | Montpellier | Surgical ICU | Philippe Burtin |
| Marne La Vallee | Jossigny | Medico-surgical ICU | Pierre Garcon |
| CHU Lille | Lille | Medical intensive care unit | Saad Nseir |
| CHU de Caen | Caen | Medical intensive care unit | Xavier Valette |
| Compiegne Noyon Hospital | Compiegne | Medico-surgical ICU | Nica Alexandru |
| Cochin | Paris | Medical intensive care unit | Nathalie Marin |
| CH Pau | Pau | Medico-surgical ICU | Marie Vaissiere |
| Victor Dupouy | Argenteuil | Medico-surgical ICU | Gaëtan PLANTEFEVE |
| CH Saint Philibert | Lomme lez Lille | Medical intensive care unit | Thierry Vanderlinden |
| Beaujon | Clichy | Medico-surgical ICU | Igor Jurcisin |
| Lariboisière | Paris | Medical intensive care unit | Buno Megarbane |
| Lariboisière | Paris | Surgical ICU | Anais Caillard |
| Saint-Louis | Paris | Surgical ICU | Arnaud Valent |
| Saint Antoine | Paris | Surgical ICU | Marc Garnier |
| Louis Mourier | Colombes | Medico-surgical ICU | Sebastien Besset |
| Avicenne | Bobigny | Medico-surgical ICU | Johanna Oziel |
| Centre hospitalier de Versailles | Le Chesnay | Medico-surgical ICU | Jean-herlé RAPHALEN |
| Robert Debré | Paris | Pediatric Intensive and Intermediate Care Unit | Stéphane Dauger |
| Saint-Louis | Paris | Medical intensive care unit | Guillaume Dumas |
| Sainte-Anne | Paris | Medico-surgical ICU | Bruno Goncalves |
| CHU de Besancon | Besançon | Medical ICU | Gaël Piton |

**Germany**

| University Hospital Ulm | Ulm | Anesthesiologic Intensive Care Department | Eberhard Barth |
| --- | --- | --- | --- |
| St. Franziskus-Hospital Münster | Münster | Klinik für Anästhesie und operative Intensivmedizin | Ulrich Goebel |
| University Hospital Ulm | Ulm | IOI-Interdisziplinäre Operative Intensivmedizin | Eberhard Barth |
| Uniklinik Düsseldorf | Düsseldorf | MX01 | Anselm Kunstein |
| Universitätsmedizin der Johannes Gutenberg-Universität Mainz | Mainz | Anästhesie-Intensivstation | Michael Schuster |
| Klinikum Darmstadt GmbH | Darmstadt | Interdiszipinaere Operative Intensivstation, Klinik fuer Anaesthesiologie und operative Intensivmedizin | Martin Welte |
| Uniklinik Schleswig Holstein Campus Kiel | Kiel | Internistische Intensivstation | Matthias Lutz |
| University Hospital Würzburg | Würzburg | Klinik für Anästhesie und operative Intensivmedizin | Patrick Meybohm |
| St Vincenz | Limburg | ICU | Stephan Steiner |
| Marienhospital Aachen | Aachen | ITS | Tudor Poerner |
| Kliniken Maria Hilf | Mönchengladbach | Internistische Intensivstation I und II | Hendrik Haake |
| Charité - Universitätsmedizin Berlin | Berlin | 43i | Stefan Schaller |
| Charité - Universitätsmedizin Berlin | Berlin | 44i | Stefan Schaller |
| Charité - Universitätsmedizin Berlin | Berlin | 8i | Stefan Schaller |
| University Hospital Duesseldorf | Duesseldorf | CIA1 | Detlef Kindgen-Milles |
| Evangelisches Krankenhaus Düsseldorf | Düsseldorf | Intensivstation | Christian Meyer |
| Florence-Nightingale Krankenhaus | Duesseldorf | 32 | Muhammed Kurt |
| Charité - Universitätsmedizin Berlin | Berlin | 144i | Karl Friedrich Kuhn |
| Krankenhaus Bethanien GmbH, Solingen | Solingen | Intensivpflege Bethanien | Winfried Randerath |
| Medical Center - University of Freiburg | Freiburg | Anaesthesiologiesche Intensivtherapiestation | Jakob Wollborn |
| Städtische Kliniken Mönchengladbach | Mönchengladbach | Interdisziplinäre Intensivstation | Zouhir Dindane |
| Klinikum Konstanz | Konstanz | I01 | Hans-Joachim Kabitz |
| Elisabeth-Krankenhaus Essen | Essen | Kardiologisch-internistische Intensivstation | Ingo Voigt |
| Johanna Etienne Krankenhaus | Neuss | Station 2 | Gonxhe Shala |
| Kliniken Nordoberpfalz AG, Klinikum Weiden | Weiden | Interdisziplinäre Intensivmedizin | Andreas Faltlhauser |

**Greece**

| Sotiria Hospital | Athens | ICU 1st Department of Pulmonary Medicine Athens Medical School, National and Kapodistrian University of Athens | Nikoletta Rovina |
| --- | --- | --- | --- |
| University General Hospital Ahepa | Thessaloniki | ICU | Zoi Aidoni |
| UNIVERSITY HOSPITAL (ATTIKON) | HAIDARI | 2nd DEPARTMENT OF CRITICAL CARE | EVANGELIA CHRISANTHOPOULOU |
| GENERAL HOSPITAL OF LARISSA | LARISSA | ICU | Antonios Papadogoulas |

**India**

| Sanjay Gandhi Postgraduate Institute of Medical Sciences (SGPGIMS) | Lucknow | Critical Care Medicine | MOHAN GURJAR |
| --- | --- | --- | --- |

**Iran**

| Imam Reza | Tabriz | General | Ata Mahmoodpoor |
| --- | --- | --- | --- |

**Iraq**

| Baghdad teaching hospital | Baghdad |  | Abdullah khudhur Ahmed |
| --- | --- | --- | --- |

**Ireland**

| Mater Misericordiae University Hospital | Dublin | Department of Critical Care Medicine | Brian Marsh |
| --- | --- | --- | --- |
| Cork University Hospital | Cork | Covid ICU | Ahmed Elsaka |

**Israel**

| Hadassah University Medical Center | Jerusalem | Corona ICU | Sigal Sviri |
| --- | --- | --- | --- |

**Italy**

| Policlinico S. Orsola-Malpighi | Bologna | Terapia Intensiva Respiratoria | Vittoria Comellini |
| --- | --- | --- | --- |

**Libya**

| Askar | Suq Elkamis | MICU | Ahmed Rabha |
| --- | --- | --- | --- |
| Tripoli University Hospital | Tripoli | MICU | Hazem Ahmed |

**Mexico**

| Instituto Nacional de Ciencias Medicas y Nutricion Salvador Zubiran | Mexico City | Department of Critical Care Medicine | SILVIO A NAMENDYS-SILVA |
| --- | --- | --- | --- |

**Morocco**

| CHU Ibn Sina de Rabat | Rabat | Service de Réanimation - Institut National d´Oncologie | Abdelilah Ghannam |
| --- | --- | --- | --- |

**Netherland**

| Alrijne Zorggroep | Leiderdorp | ICU Department | Martijn Groenendijk |
| --- | --- | --- | --- |
| Radboudumc | Nijmegen | Intensive Care department Radboudumc | Marieke Zegers |
| UMC Utrecht | Utrecht | ICU departement | Dylan de Lange |
| Medisch Spectrum Twente | Enschede | Intensive Care Center | Alex Cornet |
| Canisius Wilhelmlina Ziekenhuis | Nijmegen | ICU Department | Mirjam Evers |
| Diakonessenhuis Utrecht | Utrecht | Intensive care | Lenneke Haas |
| Zuyderland Medical Center | Heerlen | Zuyderland Heerlen | Tom Dormans |
| University Medical Center Groningen | Groningen | Department of Critical Care | Willem Dieperink |

**Norway**

| Oslo University Hospital, Rikshospitalet Medical | Oslo | Department of Critical Care and Emergencies | Luis Romundstad |
| --- | --- | --- | --- |
| Haukeland University Hospital | Bergen | General ICU | Britt Sjøbø |
| Ålesund Hospital | Ålesund | Dept. Anesthesia and Intensive Care, Surgical ICU | Finn H. Andersen |
| Kristiansund Hospital Helse Møre og Romsdal HF | Kristiansund N | ICU | Hans Frank Strietzel |
| Oslo University Hospital | Oslo | Surgical ICU | Theresa Olasveengen |
| Haugesund Hospital | Haugesund | ICU | Michael Hahn |

**Poland**

| First Independent Teaching Hospital No. 1 | Lublin | II Department of Anesthesiology and Intensive Care | Miroslaw Czuczwar |
| --- | --- | --- | --- |
| Opole University Hospital | Opole | Department of Anesthesiology and Intensive Care | Ryszard Gawda |
| Military Institute of Medicine | Warsaw | COVID-19 ICU | Jakub Klimkiewicz |

**Portugal**

| Centro Hospitalar e Universitário São João | Porto | Infectious Diseases ICU | Maria de Lurdes Campos Santos |
| --- | --- | --- | --- |
| Hospital de Beatriz Ângelo | Loures | Serviço de Medicina Intensiva | André Gordinho |
| Centro Hospitalar Tráz os Montes e Alto Dour | Vila Real | D | Henrique Santos |
| Centro Hospitalar do Médio Tejo | Abrantes | Serviço de Medicina Intensiva | Rui Assis |
| Centro Hospitalar de Tondela-Viseu, EPE | Viseu | Unidade de Cuidados Intensivos Polivalente | Ana Isabel Pinho Oliveira |

**Saudi Arabia**

| Fundeni Clinical Institute | Jeddah | MICU | Mohamed Raafat Badawy |
| --- | --- | --- | --- |

**Spain**

| Hospital Universitario Río Hortega | Valladolid | UVI Polivalente y Coronaria | David Perez-Torres |
| --- | --- | --- | --- |
| Corporacion Sanitaria Universitaria Parc Tauli | Sabadell | Intensive Care Department | Gemma Gomà |
| Hospital Universitario Sagrado Corazon | Barcelona | Intensive Care Unit | Mercedes Ibarz Villamayor |
| Hospital General Universitario de Albacete | Albacete | Intensive Care Unit | Angela Prado Mira |
| Complejo Hospitalario de Segovia | Segovia | ICU Segovia | Patricia Jimeno Cubero |
| Universitario de Getafe | Getafe | Intensive Care and Burn Unit | Susana Arias Rivera |
| Germans Trias i Pujol Hospital | Badalona | General ICU | Teresa Tomasa |
| Hospital Universitario de Burgos | Burgos | UCI Burgos | David Iglesias |
| Hospital de Tortosa Verge de la Cinta | Tortosa | Unidad de Cuidados Intensivos | Eric Mayor Vázquez |
| Hospital Universitario Rio Hortega | Valladolid | Reanimación Quirurgica | Cesar Aldecoa |
| Hospital Alvaro Cunqueiro | Vigo | Servicio de Medicina Intensiva CHUVI | Aida Fernández Ferreira |
| Clínico Universitario Lozano-Blesa | Zaragoza | Unidad de Cuidados Intensivos | Begoña Zalba-Etayo |
| Hospital Universitario Río Hortega | Valladolid | Servicio de Medicina Intensiva - Unidad 2 | Isabel Canas-Perez |
| Hospital Universitario Río Hortega | Valladolid | Servicio de Medicina Intensiva - Unidad 3 | Luis Tamayo-Lomas |
| Hospital Universitario Río Hortega | Valladolid | Servicio de Medicina Intensiva - Unidad 4 | Cristina Diaz-Rodriguez |
| H. Universitari i Politècnic La Fe | Valencia | General | Susana Sancho |
| Complexo Hospitalario Universitario Ourense | Ourense | UCI CHUO | Jesús Priego |

**Sudan**

| Khartoum Bahri Hospital | Khartoum |  | ENAS M.Y ABUALQUMBOZ |
| --- | --- | --- | --- |
| Medical Military Hospital | Khartoum |  | Momin Majed Yousuf Hilles |
| Wad Medani teaching Hospital | Wad Medani |  | Mahmoud Saleh |

**Switzerland**

| Centre Hospitalier Universitaire Vaudois | Lausanne | Service de Médecine Intensive Adulte (SMIA) | Nawfel Ben-HAmouda |
| --- | --- | --- | --- |
| Clinica Luganese Moncucco | Lugano |  | Andrea Roberti |
| Spital Thurgau Frauenfeld | Frauenfeld | IPS KSF | Alexander Dullenkopf |
| Fribourg Hospital | Fribourg | Intensive Care Unit | Yvan Fleury |
| Geneva University Hospitals | Geneva | Intensive Care Unit (Service des Soins Intensifs) | Bernardo Bollen Pinto |
| Inselspital Bern | Bern | Dept of Intensive Care Medicine | Joerg C. Schefold |

**USA**

| SUNY Downstate | Brooklyn | ICU | Mohammed Al-Sadawi |
| --- | --- | --- | --- |
