# Supplementary material for: The impact of frailty on survival in elderly intensive care patients with COVID-19: the COVIP study
Source: Crit Care. 2021 Apr 19;25:149. doi: 10.1186/s13054-021-03551-3 (PMC8054503; doi:10.1186/s13054-021-03551-3)
Supplement: Supplementary file 2 — Additional file 2.: COVIP Country map; Distribution of study sites and included patients per country. The first number is the number of ICUs per country, the second the total number of included patients per country [file 13054_2021_3551_MOESM2_ESM.pdf]

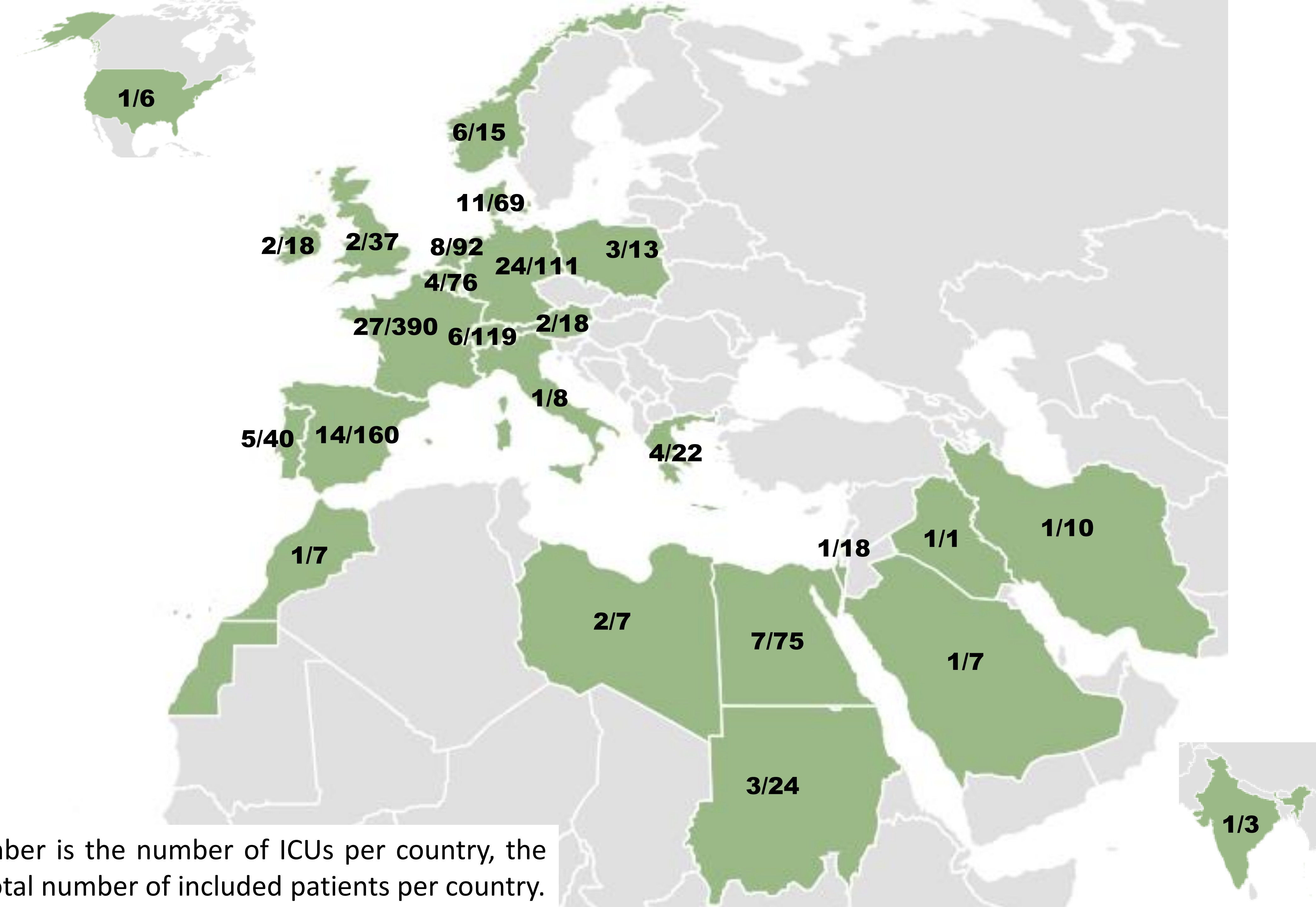

The first number is the number of ICUs per country, the second the total number of included patients per country.
