# Supplementary figures and images for: The impact of frailty on survival in elderly intensive care patients with COVID-19: the COVIP study
Source: Crit Care. 2021 Apr 19;25:149. doi: 10.1186/s13054-021-03551-3 (PMC8054503; doi:10.1186/s13054-021-03551-3)

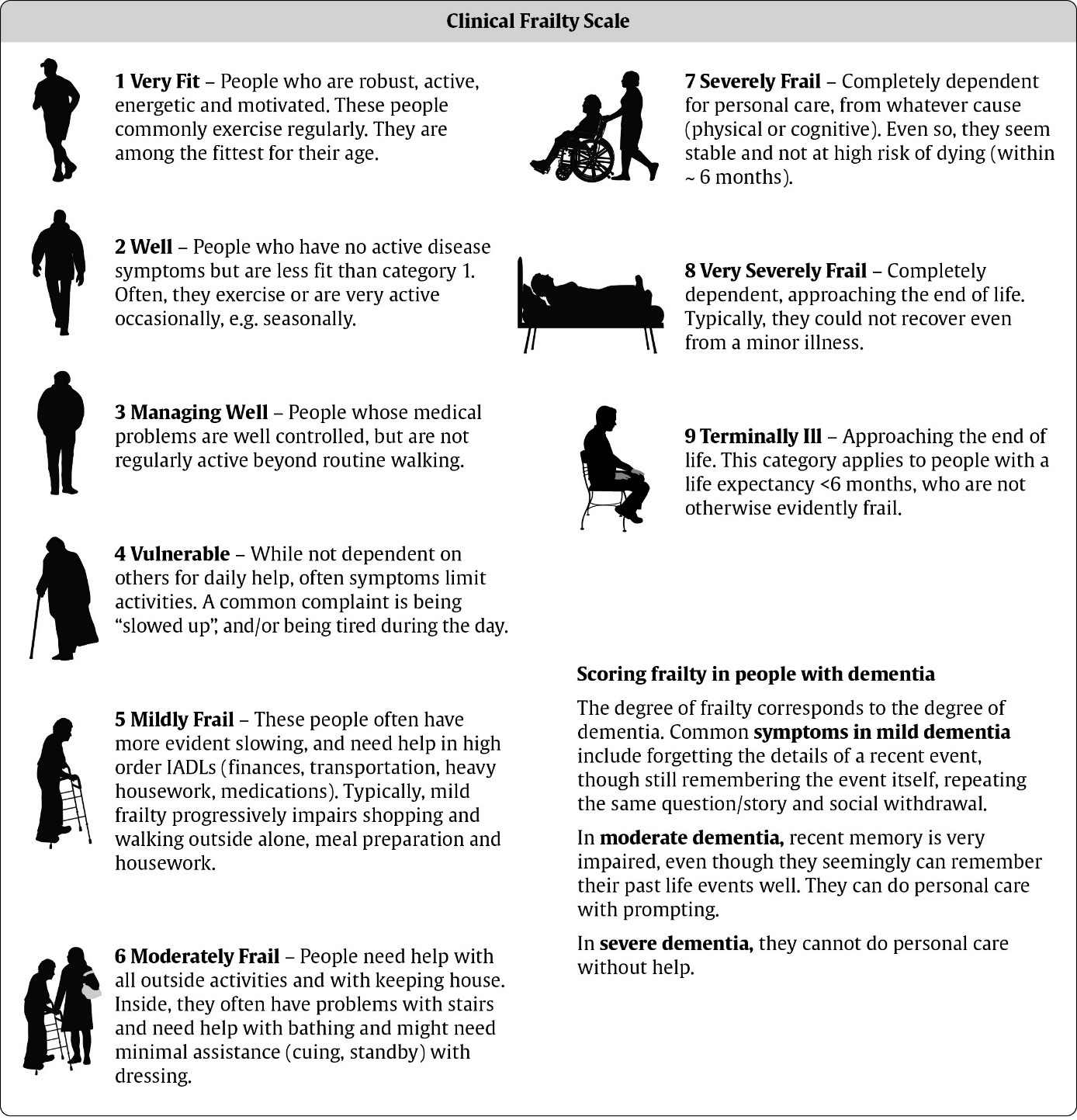


Permission to use this scale was granted from Dalhouse University

Supplement: Supplementary file 3 — Additional file 3.: Clinical Frailty Scale; Description: Pictograms and description of the Clinical Frailty Scale [file 13054_2021_3551_MOESM3_ESM.docx]

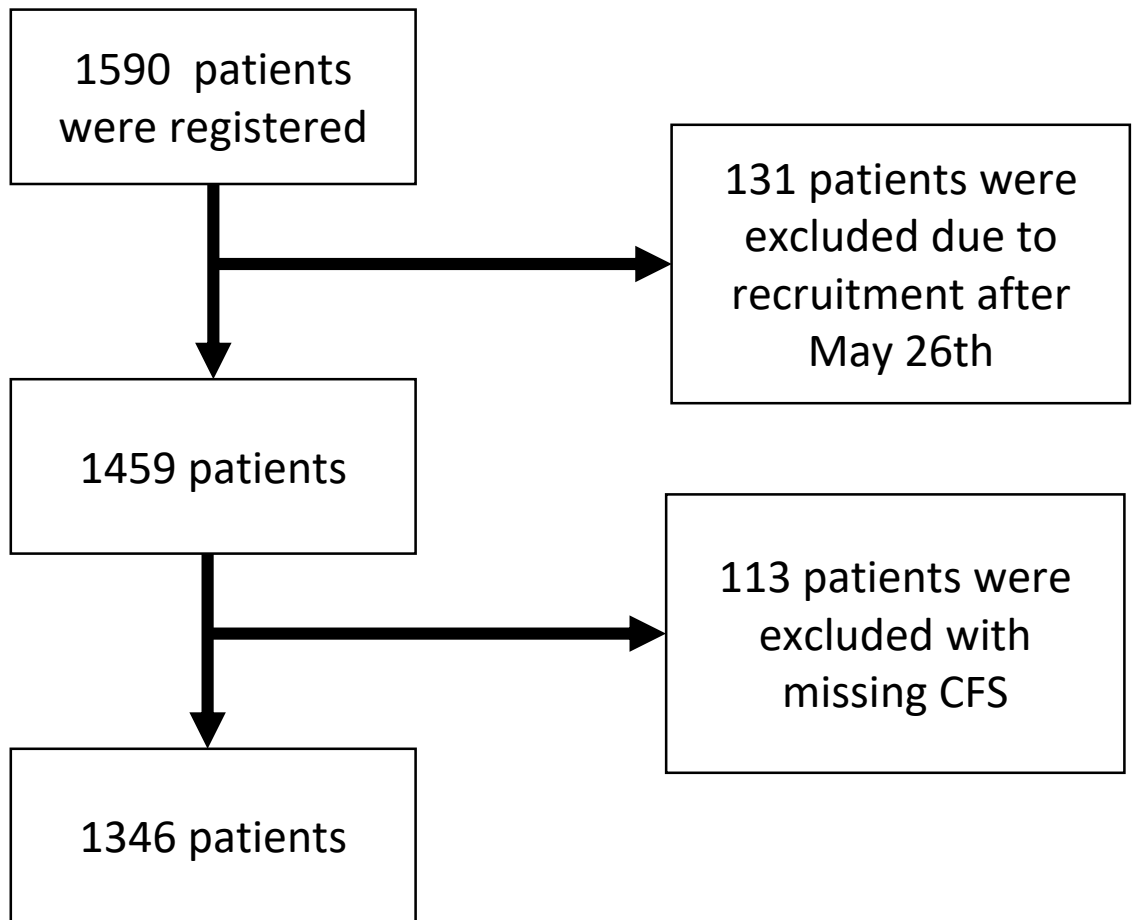

Supplement: Supplementary file 6 — Additional file 6.: Consort flow chart; Description: Consort flow chart illustrating screening and inclusion into the COVIP study [file 13054_2021_3551_MOESM6_ESM.pdf]
