# Supplementary material for: The impact of frailty on survival in elderly intensive care patients with COVID-19: the COVIP study
Source: Crit Care. 2021 Apr 19;25:149. doi: 10.1186/s13054-021-03551-3 (PMC8054503; doi:10.1186/s13054-021-03551-3)
Supplement: Supplementary file 4 — Additional file 4.: Definition of the comorbidities; Description: Detailed definition of the comorbidities of patients included in the COVIP study [file 13054_2021_3551_MOESM4_ESM.docx]

**Definition of comorbidities:**

Diabetes mellitus: documented evidence of diabetes mellitus or reported by the patient or their relatives. Prescription of anti-diabetic medication or insulin on the drug chart.

Ischaemic heart disease: documented abnormal coronary angiography, known coronary artery disease, previous percutaneous coronary intervention (PCI) or coronary bypass surgery

Chronic renal failure: documented evidence of chronic renal insufficiency Grade 3 or higher, creatinine clearance <60ml/min or chronic dialysis

Arterial hypertension: documented evidence of any grade of chronic arterial hypertension or prescription of anti-hypertensive medication.

Pulmonary disease: documented evidence of or medication prescribed for chronic pulmonary disease of any aetiology (bronchial asthma, COPD, pulmonary fibrosis), or clinical or radiological signs of chronic pulmonary disease

Chronic heart failure: documented evidence of or medication prescribed for chronic heart failure of any aetiology or echocardiographic or radiological signs of chronic heart failure.
