# Supplementary material for: The impact of frailty on survival in elderly intensive care patients with COVID-19: the COVIP study
Source: Crit Care. 2021 Apr 19;25:149. doi: 10.1186/s13054-021-03551-3 (PMC8054503; doi:10.1186/s13054-021-03551-3)
Supplement: Supplementary file 5 — Additional file 5.: Definition of organ support; Description: Definition of organ support in recruited patients to be documented in the case report form [file 13054_2021_3551_MOESM5_ESM.docx]

**Definition of organ support:**

Respiratory support: Mechanical ventilation (MV), non-invasive ventilation (NIV), prone position, tracheostomy and extracorporeal membrane oxygenation (ECMO)

Circulatory support: use of inotropes or vasopressors

Renal support: use of continuous or intermittent renal replacement therapy
