# Supplementary material for: The impact of frailty on survival in elderly intensive care patients with COVID-19: the COVIP study
Source: Crit Care. 2021 Apr 19;25:149. doi: 10.1186/s13054-021-03551-3 (PMC8054503; doi:10.1186/s13054-021-03551-3)
Supplement: Supplementary file 7 — Additional file 7.: Kaplan Meier curve illustrating survival dependent on clinical frailty scale (CFS); Description: Kaplan Meier curve illustrating survival dependent on clinical frailty scale (CFS) for each category [file 13054_2021_3551_MOESM7_ESM.pdf]

Kaplan-Meier curve illustrating survival dependent on clinical frailty scale (CFS)

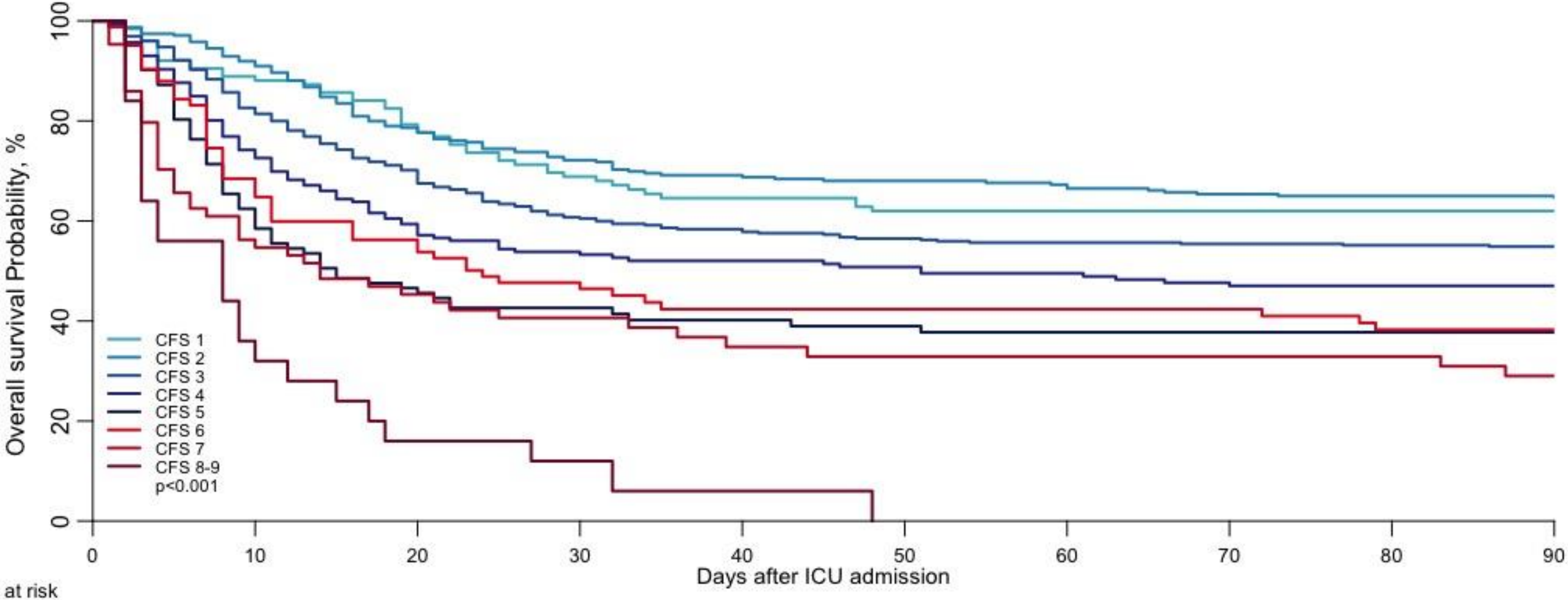

No. at risk	0	10	20	30	40	50	60	70	80	90
CFS 1	126	111	99	86	75	72	72	72	72	72
CFS 2	311	284	242	222	184	179	177	172	171	171
CFS 3	421	346	291	251	218	211	208	207	206	205
CFS 4	189	138	107	97	84	81	79	76	75	75
CFS 5	103	63	47	43	33	32	31	31	31	31
CFS 6	83	56	46	39	31	31	31	31	28	28
CFS 7	64	36	29	26	18	17	17	17	17	15
CFS 8-9	25	9	4	3	1	0	0	0	0	0
