# Supplementary material for: The impact of frailty on survival in elderly intensive care patients with COVID-19: the COVIP study
Source: Crit Care. 2021 Apr 19;25:149. doi: 10.1186/s13054-021-03551-3 (PMC8054503; doi:10.1186/s13054-021-03551-3)
Supplement: Supplementary file 8 — Additional file 8.: Numbers of deaths; Description: Numbers of deaths reported during the study/follow-up [file 13054_2021_3551_MOESM8_ESM.docx]

Numbers of deaths:

|  | ALL | Fit | Vulnerable | Frail |
| --- | --- | --- | --- | --- |
| At 30 days | 540 | 288 | 88 | 164 |
| At 90 days | 629 | 348 | 99 | 182 |
