# Supplementary material for: The impact of frailty on survival in elderly intensive care patients with COVID-19: the COVIP study
Source: Crit Care. 2021 Apr 19;25:149. doi: 10.1186/s13054-021-03551-3 (PMC8054503; doi:10.1186/s13054-021-03551-3)
Supplement: Supplementary file 9 — Additional file 9.: Survival estimates for the primary endpoint (30-day mortality) and additional time points for fit, vulnerable and frail patients as well as cumulative incidence of treatment limitations and treatment modalities Description: Table of survival estimates for the primary endpoint (30-day mortality) and additional time points [file 13054_2021_3551_MOESM9_ESM.docx]

Survival estimates for the primary endpoint (30-day mortality) and additional time points for fit, vulnerable and frail patients as well as cumulative incidence of treatment limitations and treatment modalities (95% confidence interval presented in brackets).

|  |  |  |  |  |  |  |  |
| --- | --- | --- | --- | --- | --- | --- | --- |
|  |  | **All patients** | **fit**  **(CFS 1-3)** | **vulnerable (CFS 4)** | **frail**  **(CFS 5-8)** | **p-value** |  |
|  |  |  |  |  |  |  |  |
| **Survival** | At 10 days | 78% (76-80) | 86% (84-88) | 73% (66-79) | 57% (52-63) | p < 0.001 |  |
|  | At 20 days | 65% (62-67) | 73% (70-76) | 57% (50-65) | 45% (40-52) |  |  |
|  | At 30 days | 59% (56-62) | 66% (63-69) | 53% (47-61) | 41% (35-47) |  |  |
|  | At 90 days | 52% (49-55) | 59% (56-63) | 47% (40-55) | 33% (27-39) |  |  |
|  |  |  |  |  |  |  |  |
| **Treatment limitation** | At 10 days | 24% (22-27) | 17% (15-20) | 35% (28-42) | 39% (33-45) | p < 0.001 |  |
|  | At 20 days | 32% (29-34) | 26% (23-29) | 40% (33-47) | 43% (37-48) |  |  |
|  |  |  |  |  |  |  |  |
| **Mechanical ventilation** | At 10 days | 72% (70-75) | 78% (76-81) | 68% (61-75) | 56% (50-62) | p < 0.001 |  |
|  | At 20 days | 74% (71-76) | 79% (77-82) | 68% (61-75) | 59% (53-64) |  |  |
|  |  |  |  |  |  |  |  |
| **NIV** | At 10 days | 18% (16-20) | 14% (12-16) | 21% (16-28) | 26% (21-32) | p < 0.001 |  |
|  | At 20 days | 20% (18-22) | 17% (15-20) | 25% (19-31) | 27% (22-33) |  |  |
|  |  |  |  |  |  |  |  |
| **NIV / MV** | At 10 days | 80% (78-82) | 83% (81-86) | 79% (72-84) | 70% (64-75) | p < 0.001 |  |
|  | At 20 days | 81% (79-83) | 84% (82-86) | 79% (72-84) | 72% (66-77) |  |  |
|  |  |  |  |  |  |  |  |
| **Vasoactive drugs** | At 10 days | 70% (68-73) | 73% (70-76) | 67% (60-74) | 64% (58-69) | p = 0.005 |  |
|  | At 20 days | 72% (70-74) | 75% (72-78) | 68% (61-75) | 65% (59-70) |  |  |
|  |  |  |  |  |  |  |  |
| **RRT** | At 10 days | 13% (11-15) | 11% (9-14) | 14% (9-19) | 16% (12-20) | p = 0.693 |  |
|  | At 20 days | 15% (13-17) | 15% (12-17) | 17% (12-23) | 16% (12-21) |  |  |
